# Carbon Nanotubes and Other Engineered Nanoparticles Induced Pathophysiology on Mesothelial Cells and Mesothelial Membranes

**DOI:** 10.3389/fphys.2018.00295

**Published:** 2018-03-29

**Authors:** Sotirios I. Sinis, Chrissi Hatzoglou, Konstantinos I. Gourgoulianis, Sotirios G. Zarogiannis

**Affiliations:** ^1^Department of Physiology, Faculty of Medicine, School of Health Sciences, University of Thessaly, Larissa, Greece; ^2^Department of Respiratory Medicine, Faculty of Medicine, School of Health Sciences, University of Thessaly, Larissa, Greece

**Keywords:** environmental pollutants, mesothelium, nanotoxicology, nanoparticles, nanotubes, pleura, peritoneum

## Abstract

Nanoparticles have great potential for numerous applications due to their unique physicochemical properties. However, concerns have been raised that they may induce deleterious effects on biological systems. There is accumulating evidence that, like asbestos, inhaled nanomaterials of >5 μm and high aspect ratio (3:1), particularly rod-like carbon nanotubes, may inflict pleural disease including mesothelioma. Additionally, a recent set of case reports suggests that inhalation of polyacrylate/nanosilica could in part be associated with inflammation and fibrosis of the pleura of factory workers. However, the adverse outcomes of nanoparticle exposure to mesothelial tissues are still largely unexplored. In that context, the present review aims to provide an overview of the relevant pathophysiological implications involving toxicological studies describing effects of engineered nanoparticles on mesothelial cells and membranes*. In vitro* studies primarily emphasize on simulating cellular uptake and toxicity of nanotubes on benign or malignant cell lines. On the other hand, *in vivo* studies focus on illustrating endpoints of serosal pathology in rodent animal models. From a molecular aspect, some nanoparticle categories are shown to be cytotoxic and genotoxic after acute treatment, whereas chronic incubation may lead to malignant-like transformation. At an organism level, a number of fibrous shaped nanotubes are related with features of chronic inflammation and MWCNT-7 is the only type to consistently inflict mesothelioma.

## Introduction

Nanoparticles are forms of matter with at least one dimension sized less than 100 nm. Compared to larger counterparts, they have advantageous physicochemical properties that render them suitable for a wide array of applications (Nel et al., 2006). Therefore, production and by extension disposal have markedly increased in the last decade increasing concerns with respect to workers safety, public health and the environment (Oberdörster et al., 2005). One of the most immediate routes of exposure is by inhalation, during which airborne nanomaterials may reach the distal parts of the lung and get in contact with the air-blood barrier of alveolar walls. Their potential to inflict lung morbidity is well known and has been extensively demonstrated not only in animal studies but also in epidemiological studies in humans (Fröhlich and Salar-Behzadi, 2014). On the other hand, the degree to which nanoparticles can translocate to the pleural membrane and initiate pathology remains largely unclear.

Carbon nanotubes (CNT) that occur in a fiber like shape resembling asbestos, have attracted remarkable attention. Depending on the amount of graphene sheets used to form a cylinder, they can be categorized in single and multi-walled (SWCNT and MWCNT respectively) (Sajid et al., 2016). Both of these categories are known to disrupt cellular physiology *in vitro*, which is a phenomenon that largely relies on their physicochemical properties (size, surface area, coating, adsorbance of molecules). For instance, Haniu et al. showed that three MWCNT types of distinct dimensions had differential effects on indices of oxidative stress, autophagy and plasma membrane permeability in MESO-1 cells (human pleural epithelioid mesothelioma cells) (Haniu et al., 2014). In addition, Wick et al. demonstrated that an acidic treatment is sufficient to alter the surface chemistry of CNTs and to promote the formation of rope like aggregates that are more toxic compared to well dispersed particles in MSTO-211H cells (human biphasic mesothelioma cells) (Wick et al., 2007). Apart from the influence of CNT attributes to cellular toxicity, it is also crucial to rank relevant cell types in terms of susceptibility and to clarify the existence of cell type specific effects. In that view, Hu et al. demonstrated that CNTs reduce the viability of A3 lymphocytes to a greater degree than that of MSTO-211H cells in a concentration dependent manner (Hu et al., 2010). Similarly, MSTO-211H cells were found to be more sensitive to SWCNTs than A549 cells (human lung epithelial cells) with respect to cell proliferation, cell activity, cytoskeleton organization and apoptosis. It is intriguing that alterations of the last two endpoints were observed only in the MSTO-211H cells, contrary to A549 cells that showed little to no change (Kaiser et al., 2008).

*In vivo*, Takagi et al. reported that a class of multi walled nanotubes (MWCNT-7) inflicts asbestos like pathogenicity characterized by granuloma, fibrous thickening and mesothelioma in the peritoneum of p53 heterozygous mice (Takagi et al., 2008, 2012). However, the relevance of this preliminary piece of evidence was criticized due to the use of high concentrations for the treatment of an animal model inherently susceptible to cancer. In support of the previous study, Sakamoto et al. showed that an intrascrotal injection of the same nanotubes to genetically intact rodents produced disseminated mesothelioma of the peritoneum (Sakamoto et al., 2009). Despite flaws like excessive dosage and low number of animals, this study reproduced the findings of Takagi et al. by the employment of an animal model that is not predisposed to malignancy. On the other hand, Muller et al. argued that nanotubes (with an average length of 1 μm) were not associated neither with persistent inflammation nor with carcinogenesis in the peritoneum of rats 24 months post treatment (Muller et al., 2009).

The mechanism of nanotube toxicity introduced in mesothelial cavities relies greatly on the length which is in conformity with the fiber pathology paradigm, a structure-activity relationship that emerged decades ago for materials like asbestos (Stanton et al., 1981). Poland et al. were one of the first groups to investigate the role of length in the inflammogenicity of nanotubes. Long fibrous CNTs directly injected in the peritoneum (used as a convenient surrogate of the pleura) inflicted inflammation and fibrosis of a similar or even greater magnitude compared to long asbestos fibers. However, short asbestos fibers and short CNTs produced negligible responses (Poland et al., 2008). The same authors reported comparable findings after direct installation of CNTs in the pleural cavities of mice (Murphy et al., 2011). Two factors were suggested to be crucial drivers of the proinflammatory response:(i) failure of long fibers to enter the stomatal pores leading to retention in the pleural or peritoneal cavity with a threshold of 5 μm, which was estimated by the use of precisely characterized silver nanowires (Schinwald et al., 2012b); and (ii) frustrated phagocytosis (*in vivo*, fibers >10 μm cannot be fully engulfed by macrophages and are seen protruding from the cytoplasm), that results in further recruitment of immune cells, mesothelial injury and chronic inflammation with foreign-body granuloma formation (Schinwald and Donaldson, 2012). This length dependence obtained with CNTs may also apply for other nanofibers by virtue of the fact that 20 μm nickel nanowires produced analogous responses in a mouse peritoneal model, whereas no effects were seen with <5 μm nanowires (Poland et al., 2012). On a molecular level, frustrated phagocytosis increases the production of pro-inflammatory mediators by macrophages since co-culture of THP-1 (a macrophage cell line) with long nanotubes resulted in secretion of IL-1b, TNFa, IL-6, and IL-8. In contrast, MeT5A (SV-40 immortalized human mesothelial cell line) cells remained unaffected by direct CNT exposure. It is remarkable that incubation of MeT5A with conditioned media from nanotube treated monocytes, stimulated an aggravated response to CNT exposure implicating a pathway with significant contribution to the pro inflammatory milieu of the pleural cavity (Murphy et al., 2012).

Nanotubes exist in the form of dust and can easily become airborne, rendering inhalation one of the most immediate routes of exposure (Dahm et al., 2012, 2015). Therefore, in order to initiate serosal pathology, nanotubes would have to translocate from the airways to the pleura. Ryman-Rasmussen et al. were the first to report that inhaled fibrous CNT were found embedded in the subpleural wall and within subpleural macrophages of rodents (Ryman-Rasmussen et al., 2009). Subsequent studies provided evidence in favor of the notion that these subpleural nanotubes were capable of penetrating the mesothelial lining into the pleural cavity (Mercer et al., 2010, 2013; Porter et al., 2010, 2013; Kasai et al., 2015). Based on the former data, further investigation is warranted to elucidate whether pleural deposition is associated with adverse health outcomes. Moreover, it is imperative to clarify the determinants of nanotube biokinetics and pathogenicity in pursuance of predicting their toxicological profile and designing safer materials.

Recently, female workers in a print factory under inadequate safety measures, were hospitalized due to pleural and pericardial effusions. Pathological examination of the pleura showed marked inflammatory response and fibrous thickening, that were likely to be associated with 20 nm polyacrylate/nanosilica particles that were found in samples of pleural tissues and fluids (Song et al., 2009, 2011). Due to the inappropriate workplace conditions and the lack of protective gear, the degree to which nanoparticle exposure was responsible for the aforementioned observations was debatable. That being said, the findings were replicated in a rat model of intratracheal instillation of the same polyacrylate/nanosilica, further supporting the notion that some inhaled nanoparticles may be associated with pleural pathology (i.e., effusions, inflammation, fibrosis) (Niu et al., 2016; Zhu et al., 2016).

**Figure 1:**
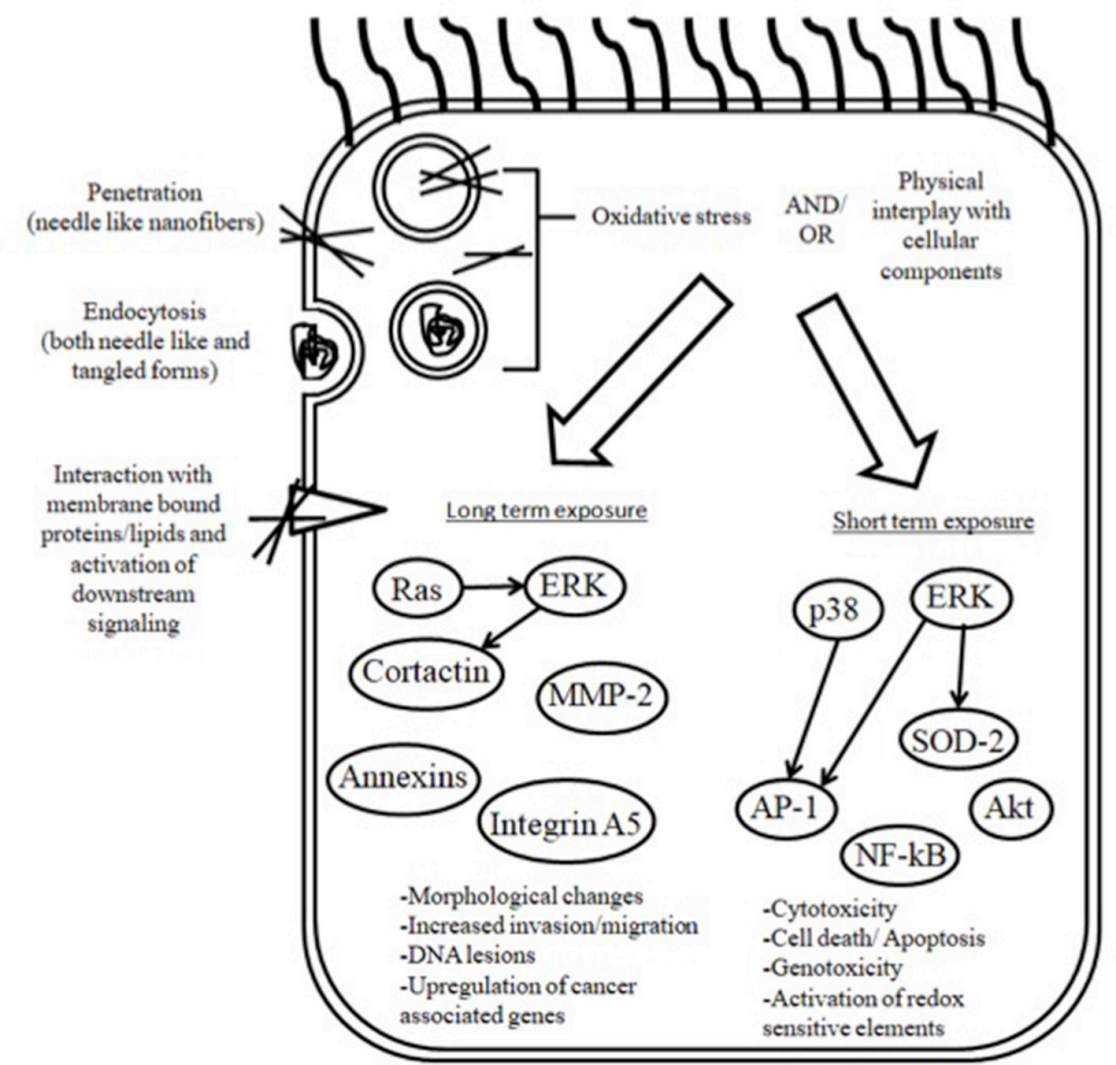
Overview of the mechanisms of cellular uptake and toxicity of CNTs in mesothelial cells.

Asbestos is well accepted to predispose chronically exposed people to a number of benign pathologies particularly asbestosis, which is a form of interstitial disease, pleurisy, pleural effusions and pleural plaques as well as malignancies including mesothelioma (Hesdorffer et al., 2008; Kim et al., 2017; Richards, 2017). Due to a delayed and inadequate policy response, the incidence of asbestos associated disorders is still considerably high (Delgermaa et al., 2011; Prazakova et al., 2014; Bibby et al., 2016). In view of the asbestos paradigm, it is crucial to evaluate the degree to which some of the newly introduced engineered nanomaterial pose a risk for serosal membrane associated pathology. The aim of this review article is to provide an overview of the studies describing nanoparticle adverse effects on mesothelial cells and membranes. Cellular uptake and genotoxicity in mesothelial cells following acute and chronic/subchronic nanotube administration are discussed (a schematic overview is shown in Figure 1). Additionally, we also demonstrate endpoints of serosal pathology in rodents following exposure via the respiratory system or by peritoneal injection.

## *In vitro* studies (Table 1)

### Cellular uptake of nanotubes by mesothelial cells: mechanism and the role of physicochemical properties

It is well documented that in order to disrupt cellular physiology, nanotubes have to come in contact with cells and subsequently undergo internalization (Nerl et al., 2011; Tsukahara et al., 2013). Given that different cells employ a distinct combination of uptake pathways, it is imperative that each type with increased risk is individually evaluated (Xia et al., 2008). However, mesothelial cells, which are prone to fiber carcinogenesis, have been studied to a limited extent. Haniu et al. demonstrated that MESO-1 cells internalize 150 nm, 8 μm MWCNTs as single fibers or aggregates by energy driven endocytosis, considering that its inhibitor Cytochalasin D significantly suppressed the process. Subsequently, some CNT were shown to injure the lysosomes in which they were located by breaking through the lipid layer (Haniu et al., 2011b). Nonetheless, it is well known that endocytosis comprises three distinct pathways and each of them may have different contribution to uptake. In that regard, Maruyama et al. showed that clathrin mediated endocytosis was the most prominent mechanism using two inhibitors that completely inhibited MWCNT-7 (60 nm, 10 μm) internalization by MESO-1 cells. Also, caveolae-dependent endocytosis and macropinocytosis were significantly suppressed by their corresponding inhibitors with a tendency for concentration dependence. Therefore, both were likely to have a complementary role (Maruyama et al., 2015). It should be noted however that recent evidence implicate the existence of multiple clathrin and caveolae independent endocytosis pathways and that the mechanism of uptake is not yet fully clarified in nonphagocytic cell types like mesothelial cells (Conner and Schmid, 2003; Doherty and McMahon, 2009). Consequently, one cannot rule out the existence of additional, rather unexplored, ways of nanotube cell entry. Pertinent to the above, the dispersant is documented to be an important parameter in association with MWCNT uptake. The same material (MWCNT-7 60 nm, 10 μm) in carboxylmethyl cellulose, 1,2-dipalmitoyl –sn–glycerol-3-phosphocholine and gelatin had an agglomerate diameter of 3.201, 2.336, and 1.442 nm respectively. In this study, the dispersant did not significantly affect any other physicochemical property indicating that differences in internalized CNTs could possibly be ascribed to the degree of aggregation. At the 24 h timepoint, flow cytometry confirmed that nanotubes well dispersed in carboxylmethyl cellulose did not enter MESO-1 cells, as opposed to nanotubes in the two other media. The latter decreased viability by 38 and 83% respectively whereas carboxylmethyl cellulose suspensions increased viability compared to control (Haniu et al., 2011a). With respect to the influence of structure, Nagai et al. observed an inverse correlation between diameter and viability, as determined by three different methods, luminescence, light absorbance, and fluorescence. Intriguingly, thin and rigid 50 nm MWCNT-7 were capable of entering the cell via mechanical membrane penetration, unlike 115, 145 nm or tangled counterparts. However, contrary to the results of previous studies, energy dependent endocytosis was not observed neither by Nagai et al. nor by Tabet et al., in spite of the deregulated cell function (Tabet et al., 2009; Nagai et al., 2011). Given that the mechanism of nanotube entry is well documented to rely on parameters like size and shape, it is possible that the test material can affect the mode of uptake thereby leading to outcome ambiguity. For instance, inflexible needle-like nanotubes like the ones used by Nagai et al. are known to enter cells via energy independent pathways whereas nanotubes forming supramolecular structures are predominantly endocytosed (Raffa et al., 2010). Additionally, the observation time of the study of Nagai et al. was 3 h, which was significantly shorter compared to that of the study of Maruyama et al. and may have therefore prevented the process of uptake from taking place to its full extent. Also, recent advancements suggest that nanoparticles do not always have to enter the cell in order to disrupt its physiology; in conformity with the hypothesis of Tabet et al., nanoparticles such as nanotubes were demonstrated to adhere to cell-surface receptors and transmembrane proteins initiating a molecular cascade. The downstream effectors may then include families of signaling molecules like focal adhesion and integrin linked kinases as well as ERK kinases (Kroustalli et al., 2013; Böhmert et al., 2015).

**Table 1:** *In vitro* studies describing CNT toxicity on mesothelial cells (minute = min, hour = h, month = mo) ^*^transferrin receptor 1.

**Ref**.	**Nanoparticle (dimensions)**	**Model**	**Concentration/duration**	**Pathophysiological findings**
Pacurari et al., 2008b	SWCNTs (0.8–2.0 nm)	Normal mesothelial/Malignant mesothelial	25 μg/cm^2^ for 120 min 25 μg/cm^2^ for 1, 2, or 4 h 0, 25, 75, 125 μg/cm^2^ for 30 or 60 min	ROS mediated p38, ERK upregulation AP-1, NF-kB activation Akt upregulation
Tabet et al., 2009	MWCNTs (12 nm, 0.1–13 μm)	MeT5A	100 μg/ml 24 h	Dispersion media affects agglomerate number/No internalization/disruption of mitochondrial metabolism
Haniu et al., 2011a	MWCNTs (150 nm, 8 μm)	MESO-1	10 μg/mL for 24 h	Tubes in gelatin and DPPC decreased viability by 38 and 83% resp./tubes in CMC neither entered nor damaged the cells
Haniu et al., 2011b	MWCNTs (150 nm, 8 μm)	MESO-1	1, 10 μg/mL for 24 h 50 mg/L	Endocytosis/vacuole penetration Less that 50% viability, 50% increase in permeability
Nagai et al., 2011	MWCNTs 2 to 20 nm, 3 μm 50 nm, 4 μm 50 nm, 10 μm 115 nm, 8 μm 145 nm, 6 μm	MeT5A, HPMCs	5 μg/cm^2^, 24 h incubation 5 μg/cm^2^, 3 h incubation	Inverse correlation between diameter and toxicity/no active internalization Thin and rigid able to penetrate membranes
Lindberg et al., 2013	SWCNTs (<2 nm, 1–5 μm), MWCNTs (10–30 nm, 1–2 μm)	MeT5A	5–80 μg/cm2 for 24, 48, and 72 h 40 μg/cm^2^ for 24 and 48 h 80 μg/cm^2^ for 48 h 1, 5, 10, and 40 μg/cm^2^ for 48 h	Internalization/viability reduction SWCNTs inflict DNA damage SWCNTs and MWCNs inflict DNA damage SWCNTs induce oxidative stress
Lohcharoenkal et al., 2013	SWCNTs (1–4 nm, 1–4 μm), MWCNTs (81 nm, 8.19 μm)	MeT5A	0.02 μg/cm for 4 mo	Increased migration, proliferation/Increased MMP-2, PLAU, STAT-3, AKT1, VEGFA expression
Haniu et al., 2014	MWCNTs 15 nm, 3 μm 80 nm, 10 μm 150 nm,8 μm	MeT5A, HPMCs	10 mg/ml for 24 h 10 mg/ml for 1 h 50 mg/ml for 24 h	Internalization of all CNT/150 nm resulted in most permeable membrane (29%) 15 nm resulted in greatest oxidative potential 150 nm or 80 nm induced autophagy
Lohcharoenkal et al., 2014	SWCNTs	MeT5A, LP9	0.02, 0.06, 0.2 μg/cm^2^ for 2 mo	Increased cell growth of MeT5A and invasiveness of both cell lines/Activation of H-Ras-ERK signaling/ERK mediated cortactin upregulation/Integrin AV upregulation
Zhang et al., 2015	SWCNTs (0.38 μm, 1.42 μm)	MeT5A	0.02 μg/cm for 4 mo	Increased migration, spindled morphology after chronic exposure
Wang et al., 2016	MWCNTs (2–20 nm tangled, 50 nm MWCNT-7, 115 nm, 145 nm)	RPMCs	10 μg/cm2	Hemoglobin or transferrin coated MWCNT-7 are internalized via TR1*, increase catalytic iron content and induce aggravated genotoxicity
Yu et al., 2016	MWCNT (110–170 nm, 5–9 μm)	MeT5A	0 to 40 μg/mL for 24 h	lysosomes contain MWCNTs/oxidative stress activates ERK1,2 that induces SOD-2 upregulation
Ju et al., 2017	MWCNT (110–170 nm, 5–9 μm)	MeT5A	10 mg/cm^2^ for 3 mo	Increased proliferation, invasion/DNA lesions/Altered expression of Annexins 1, 2, 5, 6

### Genotoxicity of nanotubes in mesothelial cells

While prone to fiber-associated pathology, mesothelial cells have been employed in a limited number of CNT genotoxicity bioassays. As a consequence, little is known about the molecular changes they may suffer from CNT exposure. In one such study, Lindberg et al. maintained that endocytosed short 10–30 nm, 1–2 μm MWCNTs and <2 nm, 1–5 μm SWCNTs reduce viability after 24, 48, and 72 h in MeT5A cells. Also, SWCNTs inflicted DNA damage at 40 μg/cm^2^ after 24 or 48 h. Moreover, both CNT classes were genotoxic at 80 μg/cm^2^ following 48 h of incubation. This effect can be explained in terms of the ability of nanotubes to inflict intracellular elevation of ROS, which are capable of chemically altering biomolecules such as proteins, lipids and DNA. Production of oxidative stimuli is either due to Fenton reactive metal contaminants or interaction of CNTs with biological systems, given that pure nanotubes are inherently ROS scavengers in abiotic conditions (Møller et al., 2014). However, in this study only SWCNTs were found to produce traceable amounts of malondialdehyde, a second messenger of oxidative stress, by the malondialdehyde DNA adduct assay at 1, 5, 10, and 40 μg/cm^2^ after 48 h (Lindberg et al., 2013). Therefore, it cannot be excluded that under the specified experimental parameters a significant portion of MWCNT induced DNA lesions was mediated by nonoxidant pathways (Nymark et al., 2014). For instance, disruption of cytokinesis during cell division has recently been designated as an important mechanism by which nanotubes can promote the formation of polyploid cells with altered expression of various genes including cancer-related categories (Asakura et al., 2010; Yasui et al., 2015). Also, it is known that due to similarities with microtubules of the mitotic spindle, CNTs are incorporated in the chromosomal centromere thereby promoting the formation of DNA lesions. Taken together, a portion of genotoxicity is likely to be a consequence of direct physical contact between nanotubes and cellular components (Shvedova et al., 2012). In support of this contention, Pacurari et al. reported that 81 nm, 8.19 μm MWCNT with low iron content did not significantly affect indices of oxidative stress despite inflicting cytotoxicity and DNA damage in mesothelial cells. On the other hand, the 0.8–2.0 nm wide SWCNTs induced dose dependent DNA damage at 25 or 50 μg/cm^2^ after 24 h secondary to elevated intracellular ROS levels. Intriguingly, both CNTs upregulated the redox sensitive AP-1 and NF-kB transcription factors and their upstream regulators p38 and ERK, regardless of the noticeable discrepancy in oxidative stress production (Pacurari et al., 2008a,b). In another study, Yu et al. demonstrated that a type of MWCNT (110–170 nm, 5–9 μm) elevated intracellular ROS dose-dependently and that 40 μg/ml elicited a 1.7-fold increase in MeT5A cells after 24 h. Consistent with Pacurari et al., they confirmed that ROS was an inducer of ERK that, in their study, triggered upregulation of the antioxidant enzyme superoxide dismutase 2 on an mRNA/protein level (Yu et al., 2016). Taken together, acute exposure of mesothelial cells may lead to DNA lesions and changes in molecular signaling owing to a combination of oxidant and non-oxidant mechanisms largely dependent on the nanotube class.

The short term consequences of nanotube exposure are useful in identifying cellular hazard. However, a more representative *in vitro* platform should incorporate chronic incubation at sublethal concentrations in order to shed more light to the array of molecular events that may lead to disease. In one of such studies, Lohcharoenkal et al. reported that incubation with 0.02 μg/cm^2^ SWCNTs (1–4 nm, 1–4 μm) and MWCNTs (81 nm, 8.19 μm) for up to 4 months induced proliferation, migration and invasion similar to that of asbestos fibers. By the use of qtPCR, it was observed that MMP-2 mRNA expression increased 51-fold with SWCNTs and 23-fold with MWCNTs. In conformity with the qtPCR results, MMP-2 activity was also found to be upregulated at the protein level by three different methods, Western blotting, gelatin zymography and immunofluorescence. The key functional role of MMP-2 in CNT induced aggressive phenotypes was revealed with knockdown of MMP-2 by short-hairpin RNA plasmid, which reduced invasion (60–90%) and migration of MeT5A cells (Lohcharoenkal et al., 2013). In a different study, the same group reported that treatment with SWCNTs (0.38 μm, 1.42 μm) of up to 0.2 μg/cm^2^ for 2 months increased cell growth of MeT5A cells and invasiveness of MeT5A and LP9 (human peritoneal mesothelial cells). It was then found that Ras is significantly upregulated in SWCNT exposed cells and that siRNA downregulation of Ras markedly mitigated their malignant like transformation. Amongst the H-Ras downstream targets, ERK1/2 was illustrated to be an important effector given that an ERK inhibitor suppressed invasion activity of treated cells in a dose dependent manner. Additional investigation revealed that cortactin and integrin alpha V were both overexpressed in cells incubated with CNTs and that cortactin was a downstream target of the H-Ras/ERK cascade (Lohcharoenkal et al., 2014). Recently, the phenotype of the former experiments was also verified via a physiologically relevant microfluidics based system, which monitors the migration of CNT exposed MeT5A cells under fetal bovine serum concentration gradients. Indeed, incubation with 0.02 μg/cm^2^ SWCNTs (0.38, 1.42 μm) and asbestos for 4 months resulted in a change from a flattened to a spindled shape as well as a substantially augmented migratory activity compared to untreated controls (Zhang et al., 2015). In the latest and corroborating chronic evaluation by Ju and co-workers, altered morphology, increased proliferation and invasion of MeT5A were secondary to MWCNT (110–170 nm, 5–9 μm) exposure at 10 mg/cm^2^ for 3 months. While cytotoxicity was negligible, DNA lesions were detected as early as 24 h and increased with exposure time until the end of the follow up period. Furthermore, Annexins 1 and 5 were found upregulated in MeT5A by western blotting at 30 and 90 d of incubation. On the other hand, Annexins 2 and 6 decreased by 30 d and markedly increased at approximately the 90 d timepoint. Subsequently, Annexin 1 was validated to contribute in cell migration, as siRNA knockdown significantly reduced the migratory potential of transfected MeT5A cells in a wound healing assay (Ju et al., 2017). In summary, CNTs, similarly to asbestos fibers, were demonstrated to induce malignant-like behavior in chronically exposed mesothelial cells that was driven by a gene signaling network involving H-Ras/ERK, MMP-2, Cortactin, Integrins and Annexins 1, 2, 5, and 6.

### Role of the protein corona to nanotube induced mesothelial injury

Numerous studies report that nanoparticles are capable of binding surrounding biomolecules on their surface thereby forming a layer referred to as the protein corona. Its composition, which relies greatly on the particle surface properties, is well documented to affect a number of its biointeractions (Khang et al., 2014; Ge et al., 2015; Treuel et al., 2015). Unfortunately, the role of coating constituents in nanotube associated serosal toxicity is currently limited. In that regard, Wang et al. performed an extensive mapping of absorptive proteins, amongst which hemoglobin and transferrin were further studied. Coated 50 nm MWCNT-7 produced greater cytotoxicity compared to pristine counterparts and tangled CNT incubated with hemoglobin or transferrin. Also, treatment with coated CNTs resulted in a higher intracellular catalytic iron content that was significantly correlated with increased oxidative stress levels and aggravated DNA damage. The pronounced response to nanotube-protein conjugates was driven by cellular transferrin receptor 1, given that knockdown by a specific inhibitor reduced uptake (by 20%) and cellular ferrous content. Taken together, two proteins of the corona, which is formed by biomolecules of a lung microenvironment, were shown to potentially be important players of the intricate mechanism leading to mesothelial injury, genotoxicity and by extension carcinogenesis (Wang et al., 2016). Consequently, it would be prudent to elucidate the effects of other surface-adsorbed molecules in pursuance of further understanding the complex interplay between CNTs and biological systems.

### Molecular mechanisms by which nickel impurities facilitate serosal pathology

As previously discussed, MWCNT associated toxic effects can partly be attributed to metal impurities (van Berlo et al., 2012; Aldieri et al., 2013). For instance, nickel nanoparticles (NiNPs) may unintentionally contaminate nanotubes when used as catalysts during production or they may even be added intentionally for the purpose of functionalization (Abdel Fattah et al., 2016; Yahyazadeh and Khoshandam, 2017). Given that some MWCNTs can translocate to the pleura serving as a vehicle for nickel compounds, it is important to model the events following contact of NiNPs with the mesothelial lining. To this end, Glista-Baker et al. investigated the effects of NiNPs to PDGF (platelet derived growth factor) signaling in normal rat pleural mesothelial cells. The research group showed that sublethal concentrations of either NiNPs or PDGF, which is implicated in pleural fibrosis and mesothelioma, increased mRNA and protein levels of the pro-fibrogenic CCL2 (monocyte chemoattractant protein) chemokine. Intriguingly, incubation with both NiNPs and PDGF produced a significantly aggravated response by 24 h compared to incubation with PDGF or NiNPs alone. Similarly, CCL10 (IFN-inducible chemokine) mRNA and protein levels were synergistically increased at 48 h after the combined treatment. It was then shown that PDGF mediated phosphorylation of ERK waned by 24 h, whereas the response to PDGF + NiNPs remained statistically significant. Also, pretreatment with an antioxidant, reduced ERK phosphorylation and levels of CCL2 and a MEK inhibitor attenuated the increase of CCL2 and CXCL10. Therefore, the authors suggested that NiNPs interact with PDGF signaling thereby enhancing the expression of its downstream chemokines via a mechanism involving ROS and prolonged ERK activation (Glista-Baker et al., 2012).

### The influence of nanoparticles on mesothelial layer permeability

The mesothelium is a cellular barrier with significant contribution to maintaining homeostasis in serosal cavities. Amongst its functions are the transport of fluid and particulate matter across the serosal membranes, leukocyte recruitment in response to inflammatory mediators, synthesis of pro-inflammatory cytokines and antigen presentation (Mutsaers, 2002). Silica nanoparticles were found in the pleural fluids and in the pleural mesothelial cells of occupationally exposed workers and were implicated in the induction of pleural effusions, fibrosis and granuloma (Song et al., 2009, 2011). While inflammation may have primarily accounted for the increased production of fluid, it is also important to identify whether some nanoparticles have a direct disruptive effect to serosal permeability provided they come in contact with the mesothelial cells. In a preliminary study, Arsenopoulou et al. investigated the effects of two silver nanoparticle types on the permeability of sheep parietal pleura, primary sheep pleural cell monolayers and MeT5A cells. Pre-incubation of the sheep pleura specimens with 2 μg/ml of 20 nm particles for 30 min resulted in a significant reduction of the spontaneous transmesothelial electrical resistance, an inverse surrogate of permeability. In contrast, the same concentrations of 20 and 60 nm particles increased transmesothelial electrical resistance of primary sheep pleural cell monolayers after 24 h. Similarly, MeT5A apically exposed to both NPs displayed a significant increase in transmesothelial electrical resistance by 24 h, despite being more resistant than sheep cells presumably due to interspecies differences. It is intriguing that NPs located basolaterally had a more pronounced effect on primary sheep pleural cells compared to NPs placed apically (Arsenopoulou et al., 2017). A pathway that could contribute to aberrant barrier function would be nanoparticle induced cell death, given that transepithelial electrical resistance (the equivalent of transmesothelial electrical resistance for epithelial layers) is known to be closely correlated with well-established viability indices (Konsoula and Barile, 2007). However, a more plausible explanation could be due to the disruption of tight junction integrity modulation (occludin, claudin-5, junctional adhesion molecules, zonula occludens-1) as recent studies suggest regarding sublethal doses of a number of NPs. Such a phenomenon has currently been demonstrated in epithelial monolayers of intestinal cells or blood-brain barrier models whereas no mechanistic data are available specifically for mesothelial membranes, which require further research (Dan et al., 2015; Li et al., 2015; Williams et al., 2016; Imai et al., 2017).

## Pleura (Table 2)

### Pleural pathology after exposure through the respiratory system

Murphy et al. were one of the first groups to study the role of length in nanotube associated respiratory morbidity following exposure though the airways. By pharyngeal aspiration they exposed C57BL6/J mice to 25 μg of long (165 nm, 36 μm), short (25.7 nm, 1–2 μm) and tangled (15 nm, 1–5 μm) MWCNT classes. Subsequently mice were euthanized at 1 and 6 weeks and their pleural cavity was lavaged to investigate the inflammatory response. At the 1 w timepoint only long CNT were associated with a small but significant increase in granulocyte content. By 6 w there was a marked elevation in total cell counts, LDH and protein levels owing to long CNT exposure. Pathological examination of the parietal pleura revealed lesions characterized by foreign body giant cells, granulocytes and collagen deposition. Moreover, CNTs were identified in sections of the diaphragm of long CNT treated mice, which was suggestive of the ability of long fiber-like nanotubes to translocate to the pleural cavity (Murphy et al., 2013).

**Table 2:** CNT associated serosal toxicity after respiratory system exposure (day = d, week = w, month = mo, year = yr).

**References**	**Nanoparticle**	**Model**	**Concentration/duration**	**Pathophysiological findings**
Poland et al., 2008	MWCNT 20–30 nm, 0.5–2 μm 15 nm, 1–5 μm 15 nm, 5–20 μm 40–50 nm,13 μm 20–100 nm, max. 56 μm	C57BL/6J mice (intrapleural injection)	5 mg for 24 h, 7 d, 4 w, 12 w, and 24 w	Inflammatory response to long CNTs (13 μm, max 56 μm)/lesions characterized by inflammation, mesothelial proliferation, fibrosis and CNT aggregates at 24 w
Mercer et al., 2010	MWCNT (49 nm, 3.9 μm)	C57BL/6J mice (pharyngeal aspiration)	10, 20, 40 and 80 mg per lung up to 56 d	0.6% of MWCNT burden reach the subpleura/80 mg resulted in increased penetrations at days 1, 28, and 56
Porter et al., 2010	MWCNT (49 nm, 3.86 μm)	C57BL/6J mice (pharyngeal aspiration)	80 mg up to 56 d	MWCNTs penetrated the pleura/Pleuritis in one mouse exposed to 10 g, all mice exposed to 20 or 40 g, and three mice exposed to 80 g
Xu et al., 2012	MWCNT-7 MWCNT-N	F344 rats (intrapulmonary spraying)	5 sprayings of 250 mg for 9 d	Pleural deposition/Hyperplasic proliferative lesions of the visceral mesothelium
Mercer et al., 2013	MWCNT	C57BL/6J mice (inhalation)	5 mg/m^3^ for 5 h/d for 12 d	At 336 d MWCNT were found in chest wall and diaphragm
Murphy et al., 2013	MWCNT (165 nm, 36 μm)	F344 rats (intrapulmonary spraying)	25 μg for 6 w	Pleural penetrations by few nanotubes/lesions with foreign body giant cells, granulocytes and collagen
Porter et al., 2013	MWCNT	C57BL/6J mice (inhalation)	10 mg/m^3^, 5 h/d for 2, 4, 8 or 12 d	MWCNTs penetrated the pleura of two mice at 12 days post-exposure
Xu et al., 2014	MWCNT 150 nm, 8 μm 15 nm, 3 μm	C57BL/6J mice (intrapulmonary spraying)	1.625 mg for 24 w	Long CNT in pleural cavity/Long CNT more potent in inducing hyperplasic proliferative or fibrotic foci of the parietal pleura
Fujita et al., 2016	SWCNT-MWCNT (0.5 and 1.8 μm resp.)	Wistar rats (intratracheal instillation)	0.15 or 1.5 mg/kg over 90 d	Short MWCNT migrate to the pleura more efficiently and induce greater inflammation and proliferation compared to short SWCNT
Kasai et al., 2015	MWCNT-7 (90.7 nm, 5.7 μm)	F344/DuCrlCrlj rats (inhalation)	0, 0.2, 1 and 5 mg/m^3^ for 62 times in a 13 week period	Visceral pleural and subpleural inflammation/CNT located in the diaphragm
Kasai et al., 2016	MWCNT-7 (90.7 nm, 5.7 μm)	F344/DuCrlCrlj rats (inhalation)	0.02, 0.2, or 2 mg/m^3^ for 6 h/d, 5 d/w up to 104 w	With increasing dose, pleural penetrations became more frequent/Hyperplasic lesions at 2 mg/m^3^
Suzui et al., 2016	MWCNT-N	F344/Crj rats (pharyngeal aspiration)	1 mg over 14 d (109 w duration)	Benign proliferative lesions and increased mesothelioma incidence

Xu et al. further investigated the ability of fibrous MWCNT to induce pleural lesions by exposing F344 male rats via intrapulmonary spraying with 0.5 mL of 500 μg/mL suspensions containing 165 nm, 36 μm MWCNT-N (Nikkiso, Tokyo, Japan), MWCNT-7 (Mitsui-7) or crocidolite, five times over a 9-day period. At the end of the experimental period, rats were euthanized and pleural lavage and specimens of lung and chest tissue were collected. In line with Murphy et al., the cavity fluid contained increased amount of total cells, LDH and protein. Also, the authors reported evidence of pleural deposition as lavage cell pellets contained CNT and crocidolite control mostly located in macrophages. In further support of that notion, tissue samples had a small number of fibers that were found directly penetrating the visceral pleura into the pleural cavity. In addition, the visceral mesothelium of nanotube and crocidolite treated rats developed hyperplastic proliferative lesions [10 times higher proliferating nuclear antigen index (PCNA) compared to control] closely associated with inflammatory infiltrate and focal fibrotic response. On other hand, the parietal pleura showed no pathological alterations. To examine the role of inflammation in the induction of hyperplastic proliferative lesions, the authors exposed MESO-1 cells to conditioned media from CNT/crocidolite treated macrophages and to concentrated supernatants of pleural lavage. Incubation resulted in increased proliferation of mesothelial cells thereby suggesting that macrophage secreted mediators were important drivers of the response (Xu et al., 2012).

In another study, the same authors extended the experimental duration to 24 weeks to elucidate whether size and shape can influence pleural deposition and pathological features. Two groups of six F344 rats were treated with 250 μg/ml CNT suspensions by transtracheal intrapulmonary spraying once every 2 weeks, 13 times for a total of 1.625 mg/rat. Consistent with Murphy et al., needle-like 150 nm, 8 μm MWCNTs, unlike aggregated 3 μm, 15 nm MWCNTs, were located in fibrotic parietal pleura of 4/6 rats with few nanotubes penetrating the mesothelial layer. The feasibility of pleural migration was further consolidated by the observation that nanotubes, as in the previous report, were within or attached to macrophages of the lavage cell pellets. Pathological examination of tissue sections showed that long CNTs were inside fibrotic lesions significantly thicker that those in short CNT treated rats. Moreover, long CNT were able to induce mesothelial proliferation foci in the parietal pleura accompanied by a significant elevation in PCNA levels of parietal and visceral mesothelial cells. Finally, analysis of the lavage showed that long CNTs inflicted a significantly greater increase in total cell infiltrate, protein and cytokine levels of IP-10, RANTES, IL-2, and IL-18 compared to short CNT (Xu et al., 2014).

**Figure 2:**
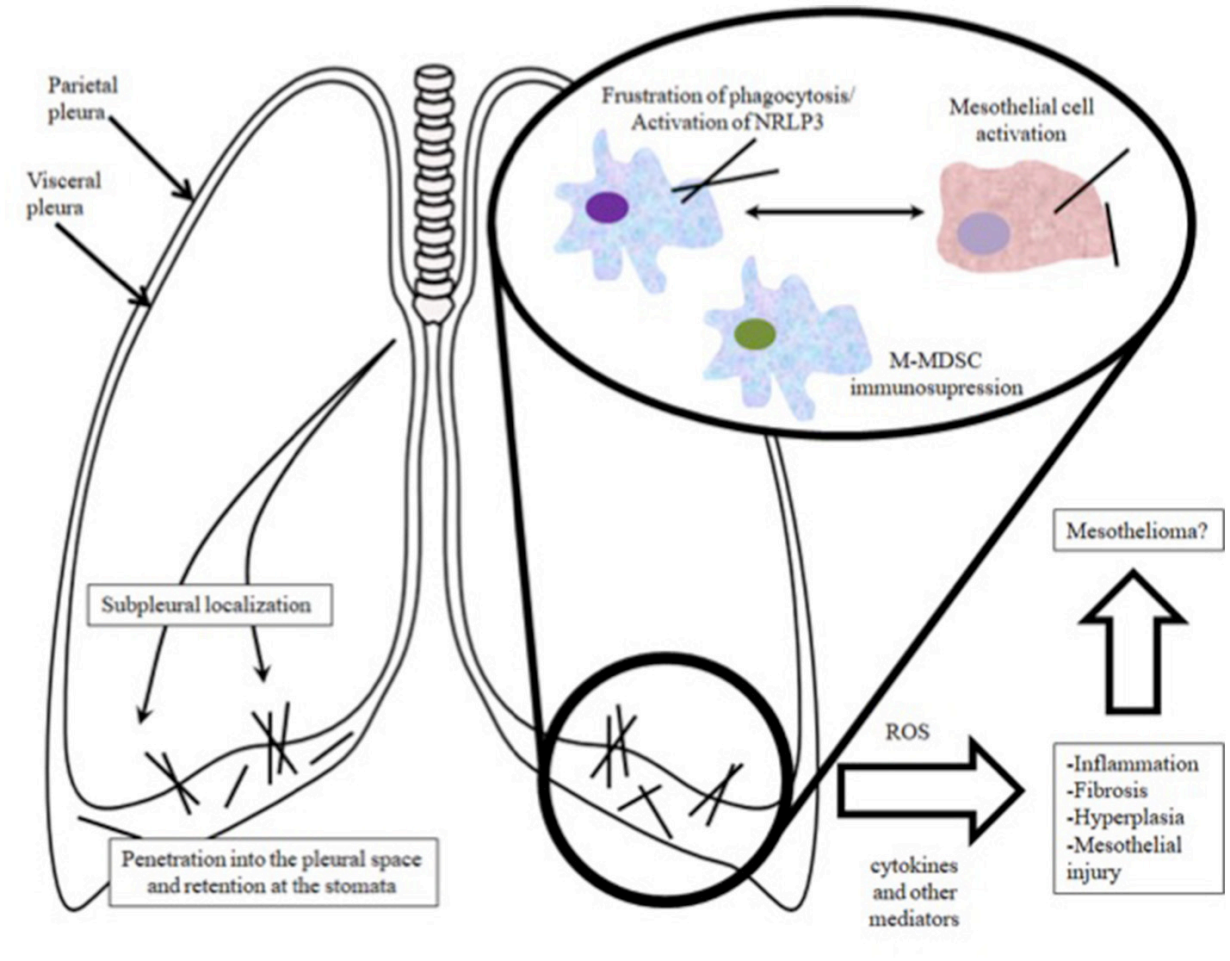
Overview of the route of entry of CNTs into the pleural cavity and the associated pathophysiological events.

In conclusion, *in vivo* experiements suggest that long, rod like nanotubes can translocate from the airways to the pleura, where they initiate inflammation with granuloma formation, that precedes focal fibrosis and hyperplasia. The immune response to nanofibers involves recognition of CNT surface or its corona by macrophage receptors (scavenger, mannose, compliment, immunoglobulin or toll-like receptors), followed by initiation of phagocytosis and upregulation of downstream cascades related to pro-inflammatory responses (Dobrovolskaia and McNeil, 2007; Gustafson et al., 2015; Sayan and Mossman, 2016). One of the major ways by which CNTs can promote tissue damage/tumor growth is stimulation of NLRP3 inflammosome to upregulate cytokine/chemokine secretion through a number of pathways and most importantly frustration of phagocytosis (O'Neill, 2008; Palomaki et al., 2011). If considered together, inflammation and direct genotoxic effects of CNTs may inflict DNA damage particularly in constituent cells of hyperplasic lesions, which more readily accumulate mutations by virtue of their increased proliferation and survival. As a result, they are thought to be the site of carcinogenesis, which gradually takes place on the condition that the pro-inflammatory milieu persists for long term (a schematic overview is shown in Figure 2). In contrast, very thin, curly and tangled tubes usually exist in the form of agglomerates that after inhalation may locate subpleurally, migrate to the pleural space and effectively undergo drainage to the lymphatics. Given that similarly to clearance, phagocytosis by macrophages remains unpurturbed, mesothelial carcinogenicity of such materials is very unlikely (Pauluhn, 2010).

According to our knowledge derived from asbestos, mesothelioma has a latency period of three to four decades, thus rendering chronic assessments indespensable in the effort to investigate the notion that CNT exposure is relevant to tumorigenesis (Toyokuni, 2014). In that context, Suzui et al. used transtracheal intrapulmonary spraying to treat F344/Crj rats with previously employed MWCNT-N (1–20 nm, 4.2 μm) 8 times over a 2-week period for a total of 1 mg. Moreover, raw CNTs were divided by a sieve in two fractions, the “flow through” (2.6 μm) and the “retained” to investigate the influence of size. After 24 h from the last administration, 5 rats from each group of 20 animals were euthanized and the amount of CNT in their lungs was measured. At the end of the experimental duration of 109 weeks, 25.4, 48.0, and 26.3% of the unfiltered, flow though and retained treatments respectively were shown to remain in the lungs. Mesothelioma of the mediastinal space with an incidence of 6/38 was observed across the board whereas none of the 28 rats of vehicle/untreated control groups showed signs of respiratory system tumors. It is intriguing that despite a non-statistically significant intergroup difference in terms of incidence, the “retained” fraction, which was considerably agglomerated, did not account for any of the pleural mesotheliomas (Suzui et al., 2016).

In contrast to the authors above, Kasai et al. exposed F344/DuCrlCrlj rats by inhalation in chambers to 0.02, 0.2, and 2 mg/m^3^ of 83.8 nm, 5.2 μm MWCNT-7 for 104 weeks, 5 days/week, 6 h/day. In the pleural lavage fluid the CNTs were present as individual, long, straight fibers with negligible aggregation and their number increased with concentration (38, 134, and 1,468 fibers in the 0.02, 0.2, and 2 mg/m^3^ male groups and 23, 240, and 847 fibers in the 0.02, 0.2, and 2 mg/m^3^ female groups). At 2 mg/m^3^ the incidence of mesothelial hyperplasia and focal fibrosis of the parietal pleura was increased in male rats. Additionally, the incidence of focal fibrosis of the ventral pleura and the incidence of mediastinal inflammation were elevated in male/female and male animal groups respectively (Kasai et al., 2016). However, as opposed to the former experiment of MWCNT-N, no evidence of pleural mesothelioma was found for chronically inhaled MWCNT-7, even though both CNTs shared similar toxicity after 9 days in a rodent model of intrapulmonary spraying.

So far, long nanotubes have attracted remarkable interest due to evidence that they exhibit asbestos-like pathogenicity. On the other hand, the consequences of short nanotube exposure and particularly SWCNT exposure in the respiratory system are rather unexplored. To address the issue, Fujita et al. divided Wistar rats into three groups of 9 animals and treated the first two with 0.15 mg/kg or 1.5 mg/kg of 0.5 μm SWCNTs and 1.8 μm MWCNTs by intratracheal instillation with the third group serving as vehicle control. In the pleural cavity lavage, MWCNTs at both doses increased macrophage count and IL-18 and SPP-1 levels by 30 and 90 days post exposure. Additionally, the high dose regimen induced a significant rise in the protein content at 90 d. However, SWCNTs had negligible effects on these endpoints, apart from a marked elevation in macrophage number 90 days after instillation. Taken together, short MWCNTs were demonstrated to initiate a more pronounced inflammatory response compared to short SWCNTs. Two findings had important implications to the mode of action; (i) short MWCNT were found penetrating the alveolar wall in tissue sections; and (ii) mediastinal lymph nodes were loaded with MWCNTs that accumulated time dependently. Hence, the authors postulated that short MWCNT either directly reached the visceral pleura or alternatively crossed through the stomatal pores and into the pleural cavity where they initiated inflammation before being cleared through the lymphatics. Intriguingly, short SWCNT were also found in mediastinal lymph nodes at 90 d, which suggests they may have followed a similar route. Discrepancies in the degree of pleural response can be explained based on the ability of short MWCNT to more efficiently migrate to the pleural cavity as compared to short SWCNT. In further support of this assertion, short SWCNTs were shown to substantially accumulate in the lungs instead, where they elicited a more pronounced effect that short MWCNT (Fujita et al., 2016).

### Nanoplatelets: a novel structure activity-relationship

As nanoparticles emerge in a vast number of shapes and sizes, there has been growing concerns that conformations apart from fibrous ones may pose a significant risk to the respiratory system. Shvedova et al. for instance, estimated by the use of aerodynamics calculations that graphene nanoplatelets are respirable on the condition that their diameter is up to 25 μm. Moreover, they showed that they inflict pleural inflammation on C57BL/6J mice after direct intrapleural installation. Rodents were injected with 50 mg of platelet suspensions and tissue as well as cavity lavage samples were collected by the end of the experimental duration. Pathological examination revealed pleural histiocytic aggregates while the lavage had increased levels of polymorphonuclear cells and MIP-1 cytokine. Remarkably, retention and frustrated phagocytosis, which are core elements of the pathobiological mechanism of fibers, were shown to be associated with the aforementioned findings. In conclusion, the authors maintained that the form of nanoplatelets could pose a new threat to the pleura (Schinwald et al., 2012a).

## Peritoneum (Table 3)

### A convenient model of mesothelium injury for serosal hazard identification

Unlike length, there is currently limited evidence in terms of the role of diameter to the hazard of nanotubes. In one such study, Nagai et al. injected rats with 1 or 5 mg of 50 nm MWCNT-7, 145 nm CNTs and 15 nm tangled CNTs that have relatively low lengths between 4 and 5 μm. After 4 weeks, 50 nm CNT induced severe peritonitis, fibrosis and mesothelal proliferation whereas 145 nm and tangled CNT treated rats only showed a mild fibrotic reaction at the deposition site. Subsequently, to identify whether inflammogenicity of the 1 month experiment correlates with carcinogenicity, the authors extended the follow up period to 1 year. At the end of the experimental duration, it was found that injection of 1 mg of 50 nm CNT induced malignant mesothelioma with a higher frequency and an earlier progression compared to injections with 1 mg of 145 nm CNT or 10 mg of tangled CNT, which did not account for any tumor. Remarkably, MWCNT associated malignancies were predominantly of the sarcomatoid type (86%), which is indicative of their highly aggressive nature, and displayed homozygous CDKN2A/2B tumor suppressor gene deletions, a typical molecular abberration of asbestos induced mosothelioma. Based on these outcomes, it was suggested that reduced diameter and high rigidity are presumably attributes of fibrous nanotubes with increased carcinogenic potency (Nagai et al., 2011). In a different study, the same authors corroborated that the previously employed tangled CNT at a dose of 10 mg does not induce mesothelioma even after 3 years post injection exposure (Nagai et al., 2013). Moreover, they reported that up to 10 mg of pristine SWCNTs elicited minimal inflammation to the peritoneum after 4 weeks (Toyokuni et al., 2015). Given that non carcinogenic tangled CNT induced negligible inflammation at the same timepoint, one can also conclude that SWCNTs are probably a low risk material in terms of mesothelial carcinogenesis.

**Table 3:** Studies of intraperitoneal injection of CNTs (day = d, month = mo, year = yr).

**References**	**Nanoparticle(dimensions)**	**Model**	**Concentration/duration**	**Pathophysiological findings**
Takagi et al., 2008	MWCNT-7	p53(+/–) C57BI/6 mice	3 mg up to 180 d	Fibrogranulomatous lesions/Atypical hyperplasia/Mesothelioma
Muller et al., 2009	MWCNT (11.3 nm, 0.7 μm)	Wistar rats	2 or 20 mg (24 month follow up period)	Limited inflammation and granuloma formation around CNTs
Sakamoto et al., 2009	MWCNT-7	F344 rats	1 mg/kg of weight	Mesothelial hyperplasia/Mesothelioma in 6 out of 7 after 37 to 40 w
Nagai et al., 2011	MWCNT-7 (50 nm)	F344-Brown Norway F1 hybrids	1 and 10 mg for 1 yr	Thin and rigid CNTs have greater mesothelial carcinogenicity/Typical lesions characterized by granulomas, fibrosis, iron deposition, mesothelial hyperplasia/tumor suppressor Cdkn2a/2b deletions
Osmond-McLeod et al., 2011	MWCNT (60 nm, 12.4 μm)	C57BI/6 mice	50 mg for 24 h and 7d	Pristine inflicted inflammation at 24 h up to 7 d and fibrosis at 7 d/Degraded CNTlong1 was less inflammogenic and fibrogenic
Takagi et al., 2012	MWCNT-7 (90 nm, 2 μm)	p53(+/–) C57BI/6 mice	3, 30, 30 mg for 1 yr	Concentration dependent mesothelioma/Hyperplasic and fibrotic lesions with granuloma and monocytes
Nagai et al., 2013	MWCNT (15 nm tangled)	F344-Brown Norway F1 hybrids	10 mg for 3 yr	Absence of mesothelioma/Granuloma without iron deposition
Rittinghausen et al., 2014	MWCNTs 62 nm, 2.13 μm 37 nm, 2.53 μm 85 nm, 2.72 μm 40 nm, 4.18 μm	Wistar rats	0.2 and 1 mg 0.6 and 3 mg 0.08 and 0.4 mg 0.05 and 0.25 mg, resp. for 24 mo	Inverse correlation between curvature and mesothelioma frequency or progression/Typical lesions and peritoneal adhesions
Toyokuni et al., 2015	SWCNT (3.83 nm/4.3 nm)	Wistar rats	0.1, 0.3, 1, 3, 10 mg for 4 w	SWCNTs caused minimal inflammation/Aggregates of the nanomaterial in the peritoneal cavity
Huaux et al., 2016	MWCNT-7 (7.1 μm, 2.8 μm)	Wistar rats	6 mg for 12 mo 2 mg for 30 d	“MWCNT-7 induced mesothelioma is facilitated by an early fiber specific accumulation of immunosuppressive monocytes”

Rittinghausen et al. examined the effect of curvature to the carcinogenicity of nanotubes by employing a high and low dose of four classes of diameter and length MWCNTs termed A (85 ± 1.60 nm, 8.57 ± 1.51 μm), B (62 ± 1.71 nm, 9.30 ± 1.63 μm), C (40 ± 1.57 nm, 10.24 ± 1.64 μm) and D (37 ± 1.45 nm, 7.91 ± 1.40 μm) of decreasing linearity. Rats that were administered MWCNT A, B, C, and D developed mesothelioma at a rate of 98% (high-dose group)/90% (low-dose group), 90/92%, 94/84% and 70/40% respectively. As for mean survival time, A, B, C, and D treated animals lived on average for 194 days (high dose group)/231 days (low dose group), 207/294, 265/415, and 585/666 respectively. For reference, the amosite asbestos group had a percentage of malignancy bearing animals of 66% with a mean survival time of 623 days. By ranking the CNTs based on the two indices of carcinogenic potential, the authors observed, in conformity with Nagai et al., that increasing linearity correlated with higher incidence and earlier progression of mesothelioma, which was predominantly of the sarcomatoid type. In addition, it should be noted that amosite and MWCNT induced mesotheliomas shared the same appearances and histological markers (Rittinghausen et al., 2014). To summarize, apart from a clear length dependence, CNT parameters like diameter, rigidity and curvature seem to be critical determinants of carcinogenic potential.

For the fiber pathology paradigm to hold, biopersistence, one of its fundamental principles apart from length and aspect ratio, has to be supported by sound experimental data. In a pertinent study, Osmond-McLeod et al. examined the role of durability (the process of simulating fiber dissolution by *in vitro* treatment with chemicals), which is closely associated with biopersistence, in the health hazard of nanotubes. Initially, the authors incumbated a panel of fibers for 10 weeks in Gambles solution, which contains electrolytes present in biological systems and has a pH close to that inside macrophages. Most tested CNT and asbestos were durable, however, a 12.4 μm class showed a gradual loss of mass (30%) that ceased after 3 weeks of incubation. Electron microscopy subsequently confirmed that the proportion of long fibers was significantly reduced compared to that of the briefly exposed CNTs. Intraperitoneal injection of the intact fraction at 50 mg induced an inflammatory response in 24 h that did not subside by 7 days and also a fibrotic response at that timepoint. Degraded nanotubes (avg. length 11.1 μm) on the other hand were associated with lesser responses. In light of the above, the authors maintained that fibrous CNTs may inflict asbestos like pathology in mice, that can be however be avoided under the condition that the material has insufficient durability (Osmond-McLeod et al., 2011).

### Monocytic myeoloid derived suppressor cells (M-MDSC): a novel component of the mechanism by which nanotubes induce mesothelioma

Huaux et al. evaluated the carcinogenic response to MWCNT-7 by treating Wistar rats with a single injection of 6 mg of long 7.1 μm CNT and short 2.8 μm CNT as well as crocidolite asbestos control. The first mesotheliomas occurred as early as 6 months later whereas most animals developed tumors within 12 months. On other hand, only one animal exposed to asbestos suffered from mesothelioma. Consistent with what previously discussed, short CNT associated mesothelioma had a lower incidence compared to that inflicted by long CNT. After clinical indications of malignancy or at the end of the observation time rats were euthanized and their peritoneal cavity was lavaged. The leukocytes of the lavage were then identified by flow cytometry, which revealed that M-MDSC were markedly elevated specifically in CNT-7 treated animals suffering from cancer. In order to further investigate the phenomenon, rats were exposed to 2 mg of CNTs or crocidolite by direct injection and were carefully monitored for up to 30 days. M-MDSC was encountered as early as 24 h and did not subside by 30 days in response to carcinogenic MWCNT-7 and asbestos treatments. On the other hand, conventional C57BL6/J mice that did not develop mesothelioma post exposure showed no indication of significant M-MDSC immunosuppression. In addition, persistent M-MDSC was observed exclusively for MWCNT-7 and asbestos in Wistar rats, whereas it was only temporarily detected after exposure to non-carcinogenic agents. Taken together, an early and sustained accummulation of fiber-specific immunosuppressive M-MDSC may facilitate carcinogenesis by hampering surveillance of tumor cells by the immune system. The authors also suggested that the specificity of M-MDSC may render this response of particular use in short term *in vivo* experiments aiming to evaluate the mesotheliomagenic potential of novel nanofibers (Huaux et al., 2016).

## Gaps, obstacles and perspectives in the field of pathophysiological effects of engineered nanoparticles

*In vitro* testing serves as a useful approach for identifying cellular hazards and elucidating potential molecular mechanisms of toxicity. However, considerable progress is required before conclusions from *in vitro* studies are reliable enough to be safely extrapolated in *in vivo* conditions. Moreover, several issues concerning the *in vivo* experimental approaches also need to be addressed. In this last part of the review we briefly summarize some important shortcomings with regards to *in vitro* and *in vivo* nanotoxicology investigations in mesothelial cells and membranes.

### *In vitro* studies

- Despite concerns and the available evidence that engineered nanoparticles and carbon nanotubes may induce serosal tissue related diseases, mesothelial cells studied to a much lesser extent than other cell types of the respiratory system in terms of nanotoxicology. The available data indicate that differences in the cell uptake/clearance and antioxidant reserves are likely to be involved in the variability of their susceptibility to injury relative to the type of nanoparticle they are exposed to. The majority of studies evaluating the toxicity of a nanomaterial focus on cytotoxicity, cytokine/chemokine production, oxidative stress and genotoxicity. However, the significance of these findings is often undermined by questionable correlation with the pathogenicity observed *in vivo*. On the other hand, endpoints such as cell proliferation, migration and morphological changes that could be investigated in order to assess the hazard for mesothelioma development have attracted significantly less attention. An unexpectedly neglected area is the evaluation of temporal gene expression changes upon exposure to a specific nanomaterial. This approach could potentially assist the development of early biomarkers of mesothelial injury and predisposition to carcinogenesis in exposed individuals.
- A very important issue that has to be addressed is the heterogeneity of experimental designs in *in vitro* studies in terms of biological endpoints, duration of exposure, nanoparticle type and dispersion method. Therefore, there is a need for the development of comparable experimental protocols that will facilitate the production of meaningful results in terms of clinical impact. For this reason evidence-based guidelines that will result in minimizing experimental errors and standardizing nanoparticle specific protocols are deemed necessary (Doak et al., 2009; Lin et al., 2012; Löndahl et al., 2014). Conventional bioassays may be subject to experimental error due to nanoparticles interference with probes, substrates, and other assay components (Park et al., 2009). In addition, dispersion methods are known to affect size distribution or even pacify nanomaterial surface and in turn modify toxicity (Kroll et al., 2009).
- Proper dosimetry is difficult to perform. In most studies, concentrations are usually deduced from viability curves and exposure times are limited to the range of hours up to a few days, making it unlikely that naturally occurring exposure is simulated. Excessively high doses at the point where cell death becomes significant may lead to confounding outcomes. Additional focus could be placed on regimens that better correlate with epidemiological information as well as subchronic/chronic follow up periods to evaluate cumulative effects.
- Studies in mesothelial and other cell types have mainly focused on characterizing nanoparticles in treatment suspensions and evaluating cellular responses to exposure. On the other hand, uptake, trafficking and elimination at the single cell level still leave many open questions. Important obstacles in evaluating the above processes are the small size of nanoparticles and their occurrence in low amounts within the target biological system. This is further perplexed by the fact that nanoparticles located intracellularly may undergo modifications (e.g., adsorption of proteins and DNA, enzymatic degradation) that can alter their toxicity (Chen et al., 2013).

### *In vivo* studies

- With respect to *in vivo* studies, there are important issues regarding the method of administration. Intra-tracheal instillation and intrapulmonary spraying are useful in that they are cost effective and convenient alternatives to more physiologically relevant inhalation approaches. However, before interpreting the results of such studies one has to consider the following: (i) Exposure is performed via bolus deliveries of test nanomaterials whereas actual exposure occurs gradually over a long period of time and probably is smaller doses; (ii) deposition to the lung parenchyma via these methods is probably heterogeneous as compared to nose-only inhalation administration which could better mimic the physiological route of breathing in rodents; and iii) the dispersion of liquid-suspended nanoparticles is often inferior to that of particles in the form of dust. Taken together, instilled CNTs, primarily at higher doses, are likely to overwhelm lung defenses and clearance mechanisms thereby producing aggravated responses that would not be seen with inhalation studies (Driscoll et al., 2000). Animal studies should refine dosing regimens to prevent overload, use well characterized asbestos controls for the biological endpoints and extend follow up periods to allow for pleural translocation and clearance. However, comparative studies of inhalation and instillation approaches have to be conducted in order to clarify potential discrepancies.
- The fiber pathology paradigm is an insightful approach to explain pathogenicity on the basis of geometry (long, thin, rigid fibers) and biopersistence. However, it does not include a complete mechanistic analysis of the complex pathway of nanotube induced adverse effects. Special emphasis should now be placed on elucidating processes such as the route and mechanism of translocation at the pleura as well as the molecular background of CNT toxicity at this site. The dependence of pleural translocation on time, dose and physicochemical nanoparticle properties requires further elucidation. One of the most challenging issues in this effort is to assess the toxicologically active fraction in the pleura, which is ideally defined by the fibers that remain in the pleural cavity evading clearance mechanisms (Donaldson et al., 2010). So far, the vast majority of studies associate mesothelial toxicity with the calculated lung burden, which is unlikely to reflect this fraction. Application of novel biochemical, imaging and analytical technologies at the stomata of parietal pleura samples may pose a key concept in revealing the early events that culminate in pleural pathology over time (Donaldson et al., 2010).
- Since the issues discussed above would require large numbers of animal studies (that will include large numbers of animals) in order to address the questions that still need to be answered, ethical concerns could arise combined with cost and time burden. Rodents do not accurately mimic human physiology and pathophysiology in terms of exposure, injury and clearance of nanoparticles. In that aspect a gradual swift to well characterized relevant human *in vitro* models, invertebrate animal models and *in silico* simulations will be needed to limit the use of high numbers of animals and to produce large volume of data needed (Braakhuis et al., 2015; Clippinger et al., 2016; Polk et al., 2016; Sharma et al., 2016; Chen et al., 2017; Gonzalez-Moragas et al., 2017).

## Conclusions

It is currently urgent that further studies are performed to elucidate the risk of nanoparticles on mesothelial cells and membranes and specifically the pleura. At the moment, only MWCNT-7 is consistently shown to induce mesothelioma whereas other classes of fibrous shaped CNT as well as silver and nickel nanowires are associated with pathological features of inflammation in rodent animal studies. In contrast, tangled/aggregated CNTs are more likely to behave like granular particles with negligible carcinogenicity.

## Methods

### Inclusion criteria

Toxicological studies reporting:

- Adequate characterization (primary size, shape, aggregation in the treatment solution, impurities…) of engineered nanoparticles.
- *In vitro* endpoints using mesothelial cell lines (cytotoxicity, apoptosis/necrosis, genotoxicity, oxidative stress).
- *In vivo* endpoints of rodent mesothelial membranes (inflammation, granulomas, fibrosis, mesothelioma).

### Search terms

- “nanoparticles AND (pleura OR peritoneum OR pericardium)”
- “nanoparticles AND (pleural OR peritoneal OR pericardial)”
- “nanotubes AND (pleura OR peritoneum OR pericardium)”
- “nanotubes AND (pleural OR peritoneal OR pericardial)”
- “nanotubes AND mesothelial”

### Literature search

Two authors (SS and SZ) performed the literature search in PubMed between August 5st and August 10th 2017. Papers not in English were excluded via the use of the corresponding filter. Eventually, the final yield was 508 unique papers amongst which 55 were eligible. Author names, publication year, nanoparticle class and dimensions, experimental model, exposure conditions, assays and results were recorded. With regards to software, Mendeley and Microsoft Word were utilized for literature management and manuscript preparation respectively.

## Author contributions

SS: Performed the literature research and wrote the manuscript; CH: Carefully read the manuscript for important intellectual content; KG: Carefully read the manuscript for important intellectual content; SZ: Conceived the review, performed the literature research and wrote the manuscript. All authors read and approved the final manuscript.

### Conflict of interest statement

The authors declare that the research was conducted in the absence of any commercial or financial relationships that could be construed as a potential conflict of interest.
